# Identification of a growth-inhibitory epitope in PfRipr5, a malaria vaccine candidate against *Plasmodium falciparum*

**DOI:** 10.3389/fimmu.2026.1724796

**Published:** 2026-01-27

**Authors:** Hikaru Nagaoka, Akihisa Fukushima, Takafumi Tsuboi, Eizo Takashima

**Affiliations:** 1 Division of Malaria Research, Proteo-Science Center, Ehime University, Matsuyama, Japan; 2 Vaccines, Sumitomo Pharma, Osaka, Japan; 3 Division of Cell-Free Sciences, Proteo-Science Center, Ehime University, Matsuyama, Japan

**Keywords:** blood-stage antigen, malaria, monoclonal Ab, vaccine, wheat-germ cell-free protein synthesis system

## Abstract

**Background:**

The *Plasmodium falciparum* Rh5-interacting protein (PfRipr) is a key component of the pentameric PTRAMP-CSS-PfRipr-CyRPA-RH5 (PCRCR) complex, which is essential for erythrocyte invasion. Antibodies against PfRipr can inhibit parasite growth, but the full-length protein is structurally complex and challenging to produce as a recombinant antigen. We previously found that a specific PfRipr fragment, PfRipr5, was the most potent antigen; however, identifying minimal functional regions within PfRipr5 is critical for improving the vaccine design.

**Methods:**

We investigated PfRipr5, a truncated fragment of PfRipr consisting of EGF-like domains 5–9, and identified the epitope recognized by the growth-inhibitory monoclonal antibody 29B11. Epitope characterization was conducted using Western blotting with cysteine-substituted mutants and surface plasmon resonance (SPR) analysis with a single-site kinetics model.

**Results and conclusion:**

The identified 20-amino-acid region represents a cysteine-associated epitope recognized by the growth-inhibitory monoclonal antibody 29B11. This study defines a growth-inhibitory epitope within PfRipr5 whose recognition is associated with cysteine integrity. These findings provide a tractable molecular entry point for dissecting PfRipr function and support epitope-focused strategies for rational design of subunit vaccines against blood-stage malaria.

## Introduction

Malaria remains one of the most devastating infectious diseases worldwide, causing nearly 600,000 deaths each year, primarily caused by *Plasmodium falciparum* infections (1). Despite ongoing efforts, the development of an effective blood-stage malaria vaccine remains a major global health challenge (2–4). A promising strategy for vaccine development involves the identification of functional antigens that elicit inhibitory antibodies capable of blocking erythrocytic parasite growth (3).

The recent Phase II trial of the RH5-based vaccine (RH5.1/Matrix-M) provided the first evidence of protective efficacy by a blood-stage malaria vaccine in humans (5). This milestone confirmed the feasibility of targeting the PCRCR invasion complex (6). However, efficacy was modest and waned over time, suggesting that a single-antigen approach may be insufficient. As an essential partner in the complex, PfRipr represents a complementary target that could broaden and strengthen protective responses when combined with RH5.

PfRipr, a large cysteine-rich protein involved in merozoite invasion of erythrocytes, has emerged as a compelling blood-stage vaccine target. It forms a multi-protein complex with RH5 and CyRPA, which is essential for parasite entry into red blood cells (7). Previous studies have demonstrated that antibodies targeting PfRipr can inhibit parasite growth *in vitro*, underscoring its potential as a vaccine antigen (7–9). However, due to its large size and structural complexity, efforts to define smaller functional regions within PfRipr that can elicit neutralizing antibody responses while preserving immunogenicity are essential. Therefore, we previously identified a truncated region of PfRipr, termed PfRipr5, which is amenable to high-yield expression and has been shown to elicit polyclonal antibodies with growth-inhibitory activity (9). Sequence analyses indicate that PfRipr5 is well-conserved among *P. falciparum* isolates, in some regions even more so than RH5, further supporting its potential as a broadly relevant vaccine target (10).

Recent studies have further advanced the evaluation of PfRipr5 as a promising next-generation vaccine antigen. Takashima et al. demonstrated that immunization with recombinant PfRipr5 induced strong antibody responses and significant growth-inhibitory activity in rabbits, highlighting PfRipr5 as a progressing and immunologically relevant target for blood-stage malaria vaccine development (11). In addition, the development of PfRipr5 vaccine candidate has progressed toward clinical-grade production. Correia et al. successfully established a GMP-compliant manufacturing process for recombinant PfRipr5, confirming its scalability and stability for future vaccine development (12). We previously produced a mouse monoclonal antibody (mAb) against PfRipr5. Surface plasmon resonance (SPR) analysis showed high-affinity interactions against PfRipr5 with dissociation constants (KD) in the nanomolar range (12). Furthermore, the mAb exhibited significant growth-inhibitory assay (GIA) activity against the *P. falciparum* 3D7 strain (12), however, its epitope is unknown.

In this study, we sought to identify and characterize the epitope within PfRipr5 recognized by the growth-inhibitory mAb and to evaluate its potential as a peptide immunogen for epitope-focused vaccine design. These findings provide critical insights into the functional landscape of the PfRipr protein and support the use of epitope-focused vaccine design strategies. Our work highlights a conserved, accessible epitope within PfRipr5, which is a promising candidate for next-generation blood-stage malaria vaccines.

## Materials and methods

### Monoclonal antibody

Mouse mAb 29B11 against PfRipr5 was purchased from BEX Co., Ltd (Tokyo, Japan) (12). Specifically, BALB/c mice were immunized three times with the wheat germ cell-free protein synthesis (WGCFS; CellFree Sciences, Matsuyama, Japan) system-produced recombinant full-length PfRipr in TiterMax^®^ adjuvant. Lymphocytes from the spleen and the lymph nodes were used to fuse with P3U1 myeloma cells to produce hybridoma cells. Finally, a hybridoma clone, 29B11 (isotype: IgG1), producing mAbs recognizing recombinant PfRipr5 was expanded, purified using a ProteinG sepharose resin (Cytiva, Tokyo, Japan; 1761802) and stored at –80˚C.

### Antigen expression

**Figure 1:**
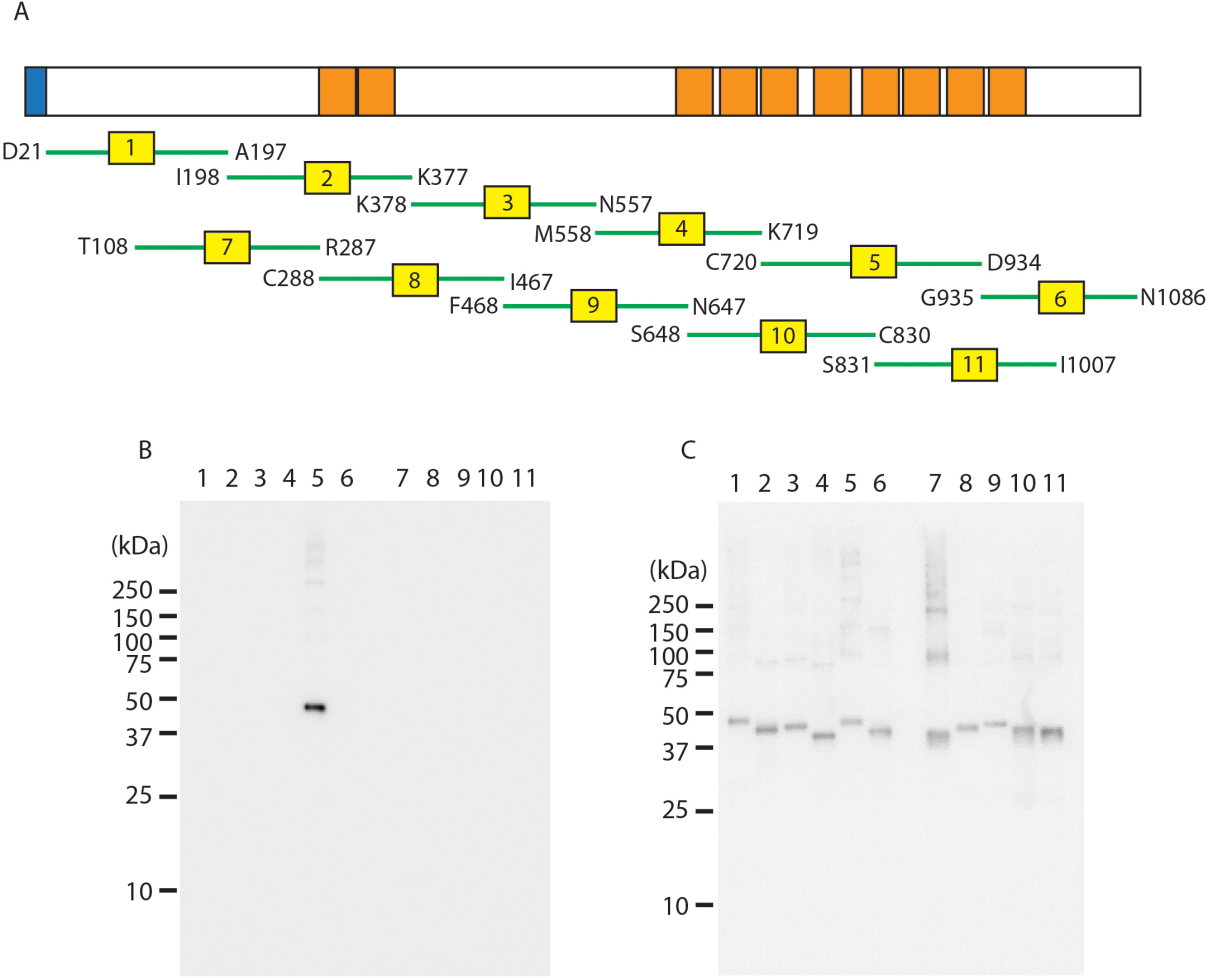
Epitope mapping of PfRipr using truncated constructs. **(A)** Schematic representation of PfRipr truncation constructs expressed using WGCFS, with EGF-like domains highlighted in orange. **(B)** Western blot analysis showing reactivity of mAb 29B11 with the PfRipr fragments. **(C)** Western blot of an independent membrane probed with an anti-GST antibody to confirm equivalent loading.

**Figure 2:**
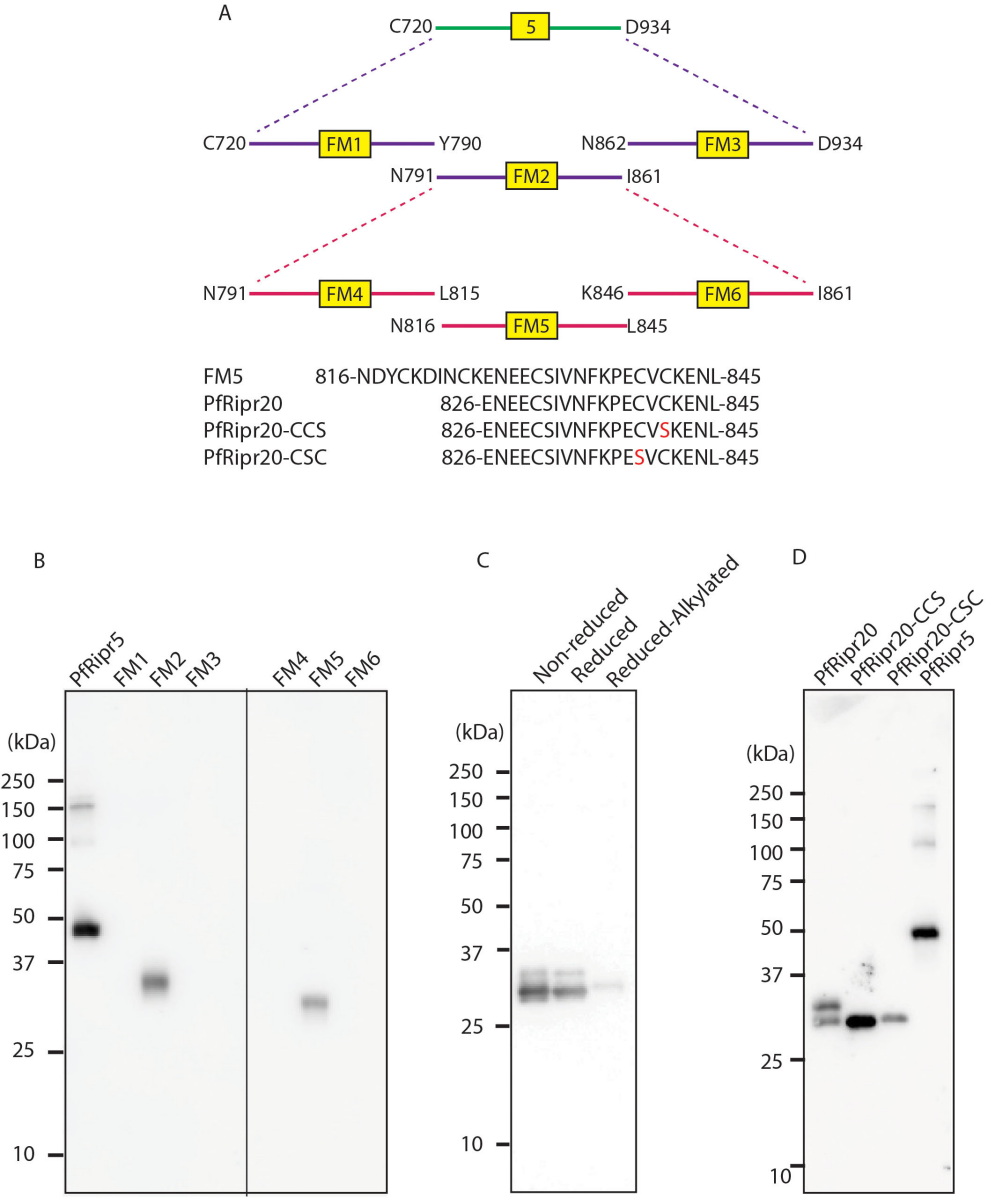
Fine mapping of the epitope of mAb 29B11 in PfRipr5. **(A)** Schematic representation of PfRipr5 fragments expressed using the wheat germ cell-free system (WGCFS). **(B)** Western blot analysis showing that fragment FM5 retained reactivity with mAb 29B11. **(C)** Western blot analysis of FM5 under non-reduced, reduced, and reduced–alkylated conditions probed with mAb 29B11. **(D)** Western blot analysis of PfRipr20 and cysteine-substituted mutants probed with mAb 29B11.

The recombinant PfRipr protein fragments (N-term GST-, C-term His-tagged) were expressed using the WGCFS system, as previously described (9). Briefly, DNA fragments encoding the PfRipr truncates were cloned into a pEU-GST expression vector (CellFree Sciences). *In vitro* transcription and translation reactions were performed using the WGCFS system (CellFree Sciences). The expressed PfRipr recombinant protein fragments were purified using a Ni2+ Sepharose affinity column (Cytiva; 17531802) (9). The amino acid sequence range of each PfRipr recombinant fragment is shown in **Figure 1A** and **Figure 2A**, including PfRipr20 protein mutants.

### Western blotting

PfRipr5 and truncated fragments were subjected to SDS-PAGE under reducing conditions and transferred to PVDF membranes unless otherwise indicated. The transfer was performed using a buffer composed of 50 mM Tris-HCl, 190 mM glycine, 3.4 mM SDS, and 20% methanol, and the final protein concentration used for the Western blot analyses was 200 nM. After blocking with 5% skim milk, the membranes were incubated overnight at 4 °C with 40.0 µg/ml of mAb 29B11, followed by 1:10,000 HRP-conjugated secondary anti-mouse IgG antibodies (Cytiva; NA931). Detection was performed using ECL (Millipore, Burlington, MA; WBKLS0500) and imaged on a LAS4000 (Fujifilm, Tokyo, Japan). Western blot analysis of the FM5 fragment was performed under non-reduced, reduced, and reduced–alkylated conditions using the FOCUS Protein Reduction and Alkylation Kit (G-Biosciences, St. Louis, MO; #786-231).

### Surface plasmon resonance assay

Binding kinetics between mAb 29B11 and GST-tagged PfRipr5 fragments were analyzed on a Biacore X100 instrument (Cytiva) at 25 °C. A CM5 sensor chip (Cytiva) was pre-coated using the Anti-Mouse Capture Kit (Cytiva; BR100838) according to the manufacturer’s protocol, the chip used in this study contained 11,694.6 RU on flow cell 1 (Fc1) and 10,014.7 RU on flow cell 2 (Fc2). Purified mAb 29B11 was captured via anti-mouse IgG, and GST-tagged recombinant proteins (PfRipr20, PfRipr20-CCS, PfRipr20-CSC) were injected at concentrations ranging from 4.7 to 75 nM in HBS-EP buffer (10 mM HEPES, 150 mM NaCl, 3 mM EDTA, 0.05% Tween-20, pH 7.4). The measurement conditions were as follows: an injection time of 120 seconds, dissociation times of 600 seconds, and a flow rate of 30 µL/min. Protein samples at varying concentrations were injected (4.7, 9.4, 18.8, 37.5 and 75 nM), starting from the lowest concentration. Surfaces were regenerated with 10 mM glycine-HCl (pH 1.7). Sensorgrams were globally fitted to a 1:1 Langmuir binding model using Biacore Evaluation software to derive kinetic parameters (k_a_, kd) and equilibrium dissociation constants (KD).

## Results

### Epitope mapping of mAb 29B11 in PfRipr using truncated constructs

To identify the epitope recognized by the inhibitory mAb 29B11, a panel of truncated recombinant PfRipr proteins covering the full-length PfRipr was expressed using the wheat germ cell-free system (WGCFS) (**Figure 1A**) as previously reported (9). We then reconfirmed that mAb 29B11 specifically recognized the PfRipr5 fragment by Western blot analysis (**Figure 1B**). An independent membrane processed in parallel was probed with an anti-GST antibody to confirm equivalent loading of the PfRipr fragments, as shown in **Figure 1C**. Additional Western blot analysis of the ectodomain PfRipr and PfRipr5 is provided in **Supplementary Figure S2A**.

### Fine mapping of the epitope of mAb 29B11 in PfRipr5

To narrow the epitope region(s) of mAb 29B11, we expressed three sub-fragments which cover PfRipr5 region designated as FM1 (C720-Y790), FM2 (N791-I861), and FM3 (N862-D934). By Western blot analyses, mAb 29B11 only recognizes FM2 (**Figure 2B**, left panel). Then, to further narrow the epitope in FM2, we expressed three subfragments that cover the FM2 region, designated as FM4 (N791-L815), FM5 (N816-L845), and FM6 (K846-I861). By Western blot analyses, mAb 29B11 only recognizes FM5 (**Figures 2A, B**, right panel). An FM5 mutant in which all cysteine residues were replaced with serine showed no detectable binding to 29B11 (**Supplementary Figure S2B**). In addition, the reactivity of mAb 29B11 was abolished when FM5 was subjected to reduction and alkylation (**Figure 2C**). Notably, residues C830 and S831 in FM5 ((NDYCKDINCKENEECSIVNFKPECVCKENL; underlined) lie at the junction between PfRipr10 and PfRipr11, neither of which was recognized by mAb 29B11 (**Figure 1B**).

To further investigate which residues contribute to 29B11 binding, we examined a shorter GST-tagged fragment, PfRipr20 (ENEECSIVNFKPECVCKENL) (**Figure 2A**), a shorter peptide containing three cysteine residues. To assess the contribution of these cysteines, we generated two cysteine-substituted variants, PfRipr20-CCS: ENEECSIVNFKPECVSKENL; and PfRipr20-CSC: ENEECSIVNFKPESVCKENL) (**Figure 2A**). All three proteins—PfRipr20, PfRipr20-CCS, and PfRipr20-CSC—were detected by mAb 29B11 in Western blotting, although PfRipr20-CCS showed stronger reactivity than PfRipr20-CSC (**Figure 2D**). This difference was further supported by densitometric quantification of the Western blot signals (**Supplementary Figure S2C**).

**Figure 3:**
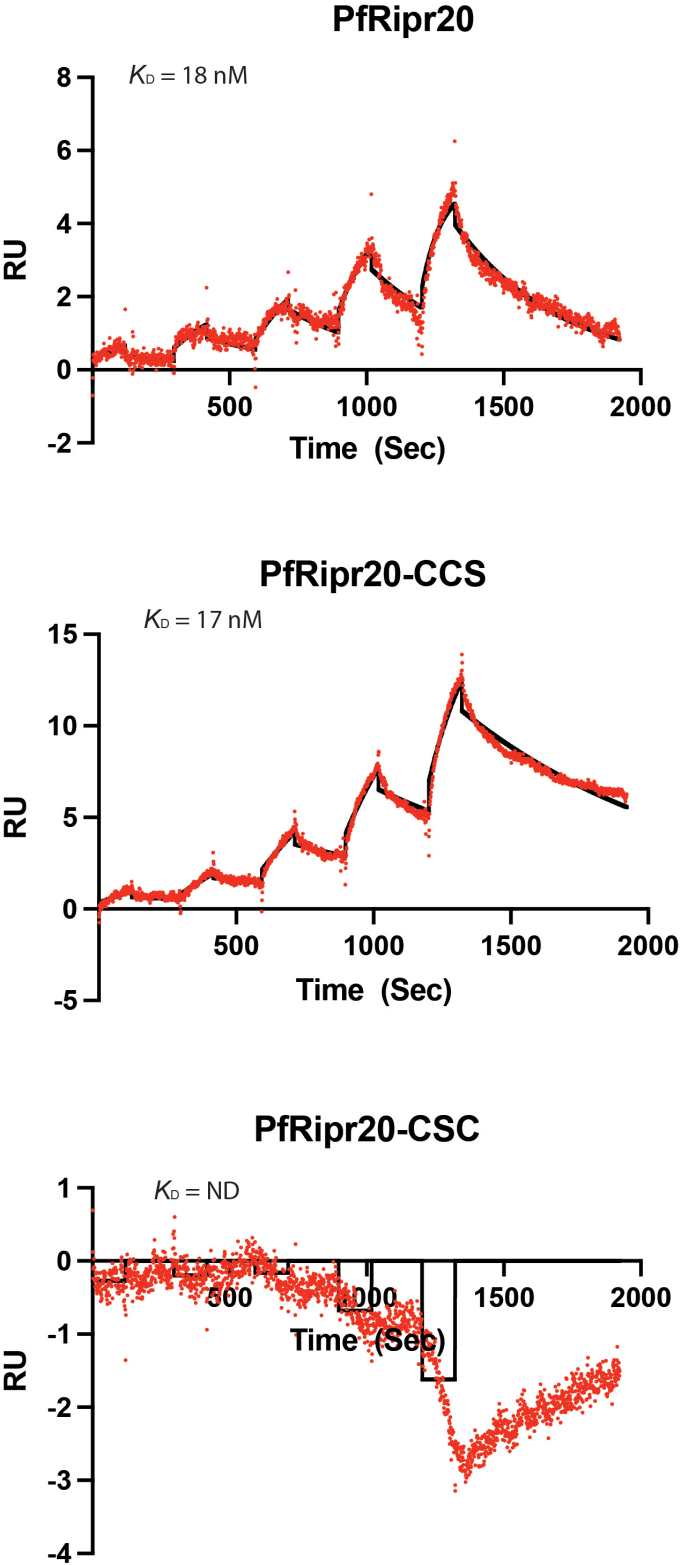
SPR analysis of mAb 29B11 with GST-tagged PfRipr20 and the mutants. Surface plasmon resonance (SPR) sensorgrams showing the binding of mAb 29B11 to GST-tagged PfRipr20 and a PfRipr20-CCS mutant, as indicated. Kinetic parameters were derived by global fitting to a single-site Langmuir binding model, and are summarized in **Supplementary Table S1**.

To quantify the protein-mAb affinities, SPR analyses were performed using PfRipr20, PfRipr20-CCS, and PfRipr20-CSC recombinant proteins against mAb 29B11. The SPR sensorgrams indicated binding of mAb 29B11 to GST-tagged PfRipr20 and the PfRipr20-CCS mutant. Kinetic parameters were obtained by global fitting to a single-site Langmuir binding model. PfRipr20 and PfRipr20-CCS bound mAb 29B11 with 16.4 ± 0.89 nM and 23.9 ± 1.78 nM KD values, respectively (n=3). In contrast, no binding of PfRipr20-CSC to mAb 29B11 was detected via SPR (**Figure 3**).

## Discussion

In this study, we mapped a functional 20-amino-acid region containing an epitope against mAb 29B11 within PfRipr5 (9, 12), a truncated region of PfRipr—a key component of the PCRCR invasion complex (5, 10, 13–15). This work highlights the feasibility of designing peptide immunogens targeting critical invasion ligands and supports the broader application of epitope-focused strategies in blood-stage malaria vaccine development.

Recent structural studies by Farrell et al. have elucidated the architecture of the RCR complex composed of PfRipr-CyRPA-RH5, showing that PfRipr comprises a multidomain core and a flexible C-terminal segment that anchors the complex to the merozoite surface through interaction with the CSS–PTRAMP complex (14). This structural arrangement highlights PfRipr’s role as a scaffold that positions RH5 and CyRPA for receptor engagement during erythrocyte invasion. In this context, our identification of an epitope in the PfRipr5 region—which spans EGF-like domains 5 to 9—adds a new dimension to PfRipr-based vaccine design. These peripheral EGF domains, although structurally distinct from the central core, are immunologically accessible and capable of inducing functional antibodies. The region encompassing PfRipr5 was poorly resolved in the recent cryo-EM structure of the RCR complex, likely due to its conformational flexibility (14). Our identification of a functional epitope within this unresolved region provides new insight into its immunological importance and potential structural organization. MAb 29B11 recognized both PfRipr20-CCS and PfRipr20-CSC mutants, although binding appeared stronger with the CCS configuration (**Figure 2D**). This suggests that the CCS pairing may better approximate the native cysteine arrangement, while reactivity to the CSC mutant could reflect cross-recognition of a similarly structured but less favored conformation. The molecular function of PfRipr has not been fully elucidated. Our epitope mapping offers a tractable entry point for dissecting functional domains within PfRipr and could lead to deeper insights into the architecture and mechanism of the RCR complex.

The recent report by Williams et al. demonstrated that antibodies associated with growth inhibition are focused within the PfRipr EGF(5–8) region (15), which is almost entirely contained within the PfRipr5 fragment used in our study (EGF5–9). This is consistent with our previous finding that full-length PfRipr immunization elicits GIA-positive antibodies targeting PfRipr5 (9). Our current study provides further refinement by showing that the monoclonal antibody 29B11 recognizes an epitope located at the center of PfRipr5, corresponding to the EGF7 domain. These data help define a core neutralizing site within Ripr and place our single-epitope mapping in the broader context of the recently described epitope landscape. The EGF78–CyRPA fusion protein vaccine (R78C) is currently being evaluated in a clinical trial (NCT07183371), and we look forward to seeing the results as they will provide further insight into the role of this region in vaccine development. In parallel, our PfRipr5, which spans a broader portion of the neutralizing epitope-rich EGF region, represents an independent and complementary approach. The present study reinforces its potential as a next-generation blood-stage vaccine antigen, and we intend to advance its further development.

According to the recently released Pf8 dataset, single-nucleotide polymorphisms (SNPs) with minor allele frequencies greater than 1% were observed within the PfRipr5 region, at A755G (6.27%) and V883L (4.77%), both of which are located outside the PfRipr20 sequence (16). Only very rare minor alleles were found within PfRipr20, with the most frequent being E843G (0.09%) and E829Q (0.05%, indicating that this 20–amino acid region is highly conserved across global P. falciparum populations. Recent deep-sequencing analyses have further shown that although PfRipr is overall more conserved than PfRH5, rare polymorphisms can still arise within functionally relevant regions. A recent preprint by Nair et al. (2025) reported SNPs in 64 of 89 Senegalese isolates, including a V840L substitution located within the PfRipr20 (16). While the functional impact of this mutation on antibody binding or growth inhibition remains unknown, such observations highlight the importance of continued genomic surveillance and functional evaluation of emerging PfRipr variants. Taken together, the high degree of conservation within PfRipr20, combined with the demonstrated functional relevance of this region, supports its potential as a promising component in future blood-stage vaccine formulations. At the same time, ongoing monitoring of PfRipr sequence diversity will be essential to ensure the robustness and durability of vaccine strategies targeting this region.

When designing vaccine formulations, it is important to maintain the correct epitope conformation, enhance its accessibility to B lymphocytes, and select a delivery method that maximizes the antigen’s structural features. Adjuvant selection is also critical to ensure proper immune activation, including cellular responses. Peptide-based vaccines offer several practical advantages, including well-defined antigen composition, ease of synthesis, reduced risk of unwanted immune responses, and improved stability. Cobaltporphyrin-phospholipid (CoPoP) liposomal formulations, which have been shown to promote durable humoral responses formulated with recombinant proteins (17, 18) and synthetic peptides (19), may provide a promising strategy to enhance the immunogenicity of PfRipr-derived epitopes. These platforms could also enable modular vaccine approaches, combining epitope-based blood-stage components with pre-erythrocytic vaccines such as R21 (20), or with peptides derived from transmission-blocking antigens like Pfs230 (19), thereby broadening and prolonging malaria protection.

In conclusion, our study identifies a cysteine-associated epitope within PfRipr5 that serves as an entry point for epitope-focused approaches in future vaccine design. The PfRipr5 region—spanning EGF-like domains 5 to 9—is highly conserved across field isolates, strengthening its potential as a broadly applicable vaccine target. Continued monitoring of sequence variation will be important to ensure the long-term efficacy of vaccines targeting this region. Future research building on these findings may help improve the design of minimal immunogens for malaria vaccines.

## Data Availability

The original contributions presented in the study are included in the article/**Supplementary Material**. Further inquiries can be directed to the corresponding authors.
