# Supplementary figures and images for: Identification of a growth-inhibitory epitope in PfRipr5, a malaria vaccine candidate against Plasmodium falciparum
Source: Front Immunol. 2026 Jan 27;17:1724796. doi: 10.3389/fimmu.2026.1724796 (PMC12886453; doi:10.3389/fimmu.2026.1724796)

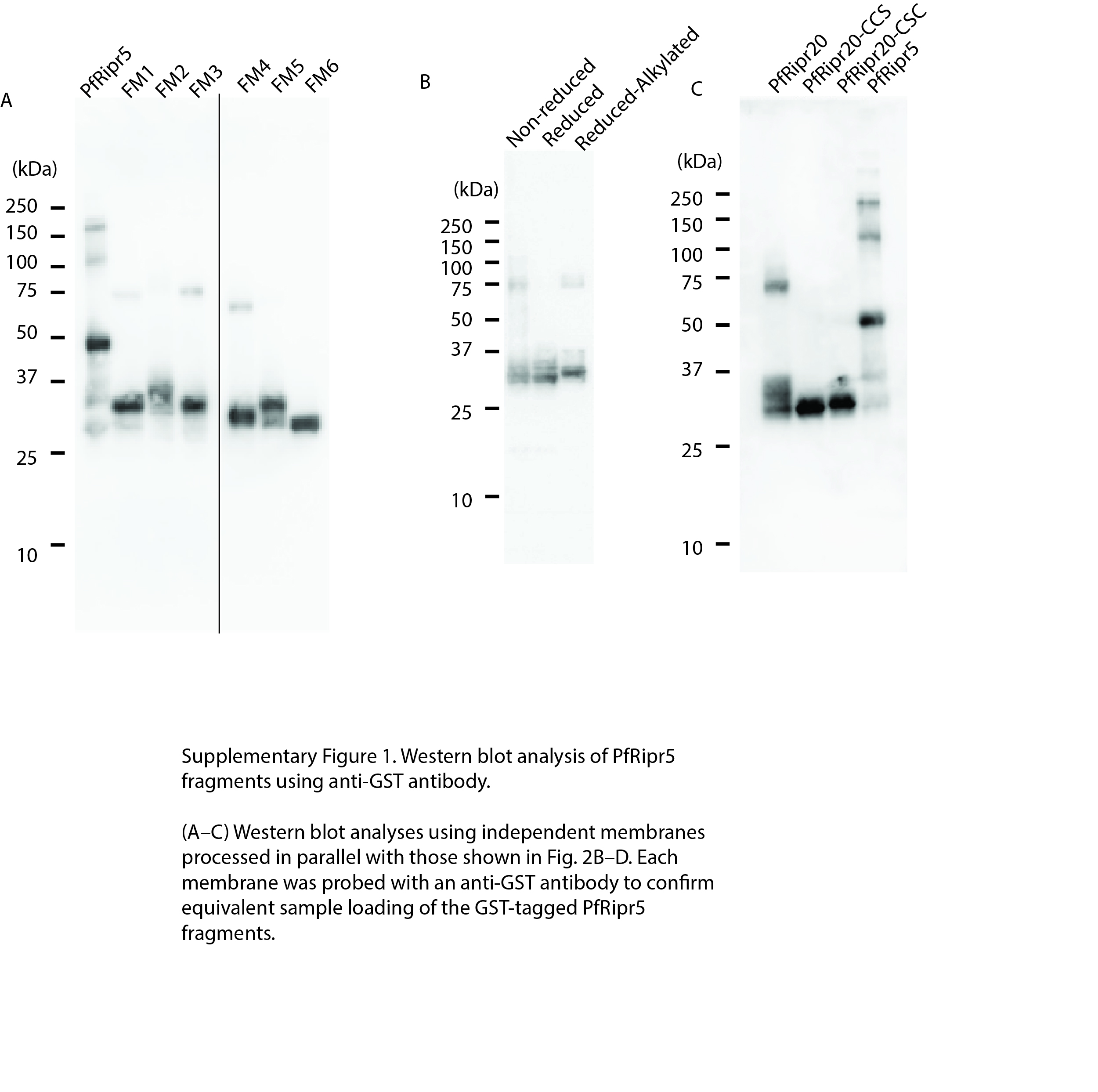

Supplement: Supplementary file 1 [file Image1.jpeg]

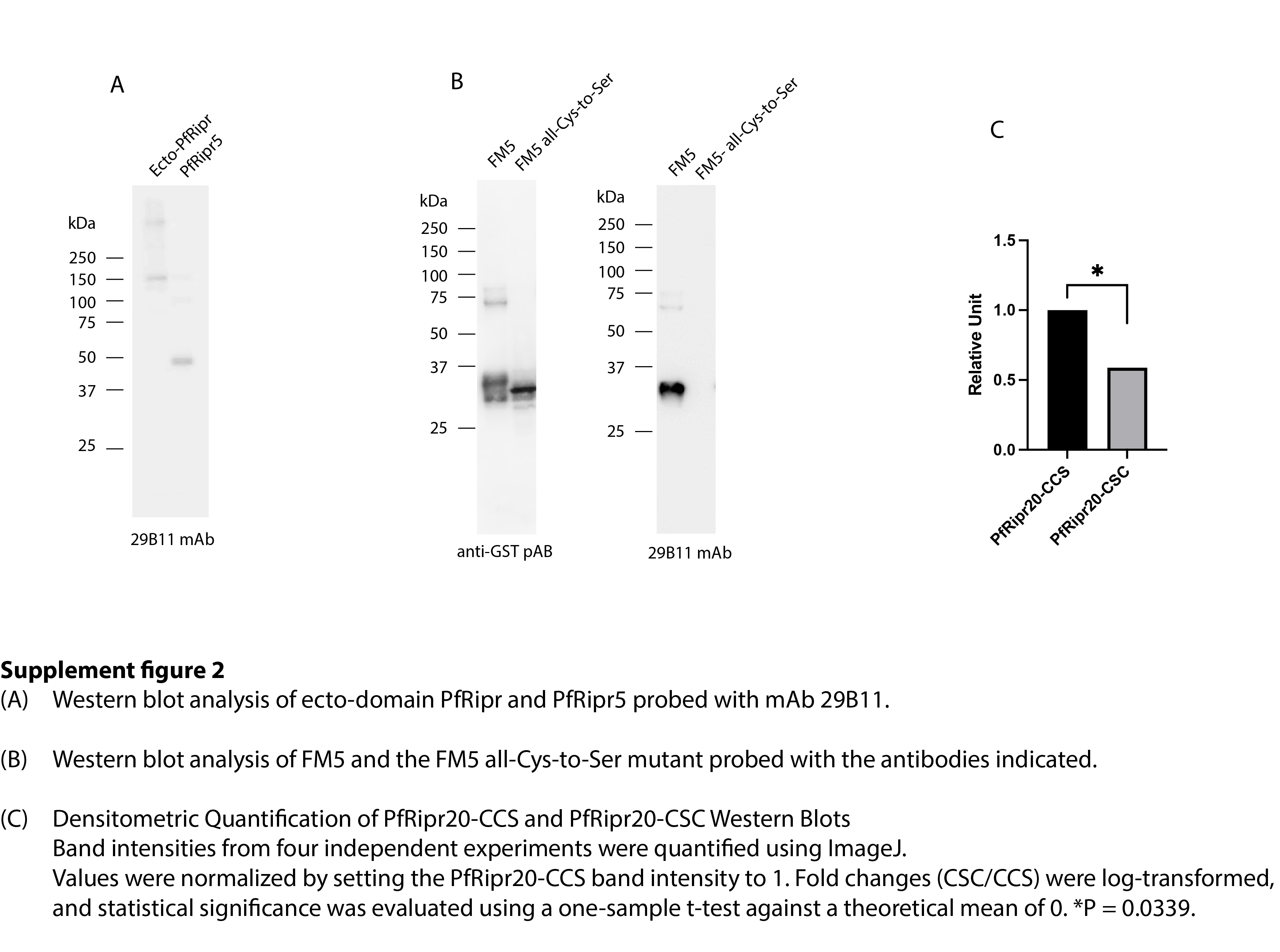

Supplement: Supplementary file 2 [file Image2.jpeg]
